# Household income and poor treatment outcome among patients with tuberculosis in Georgia: a cohort study

**DOI:** 10.1186/1471-2458-14-88

**Published:** 2014-01-29

**Authors:** Mamuka Djibuti, Eka Mirvelashvili, Nutsa Makharashvili, Matthew J Magee

**Affiliations:** 1 International School of Public Health, Tbilisi State Medical University, 33 Vazha-Pshavela Ave, 0177 Tbilisi, Georgia; 2 Department of Epidemiology, Emory University Rollins School of Public Health, 1518 Clifton Road Northeast, Atlanta, USA

**Keywords:** Tuberculosis, Prospective study, Outcome, Income, Georgia

## Abstract

**Background:**

Poverty is associated with increased risk of active tuberculosis (TB) disease onset, but the relation between household income and TB treatment outcomes is not well understood. The objective of this study was to determine household income characteristics associated with poor TB treatment outcome among newly diagnosed patients with pulmonary TB in the country of Georgia.

**Methods:**

A prospective cohort study was conducted among newly diagnosed smear positive pulmonary TB patients. Clinical and household data were collected from all consecutive patients seeking care at TB facilities in two major cities and one rural region in Georgia. Patients were followed prospectively during anti-TB regimens to determine treatment outcome. Bivariate analyses were used to determine the association of individual patient and household level characteristics with poor TB treatment outcome. A multivariable logistic model was used to estimate the adjusted association between patient household characteristics and poor TB treatment outcome.

**Results:**

After six months TB therapy, treatment outcome was available for 193 of 202 enrolled patients, of these 155 (80.3%) had a favorable TB treatment outcome. Compared to TB patients with poor treatment outcome, those with favorable treatment outcomes were younger (median 33.0 vs. 42.5 years), reported higher household monthly income (median $137 USD vs. $85 USD), were less likely to be unemployed (38.7 vs. 47.4%), and had higher level of education (38.7% vs. 31.6% with college education or greater). In multivariable analysis adjusted for age, sex, and socio-economic indicators, only low household income was remained statistically significantly associated with poor TB treatment outcome. Compared with patients from households with the highest tertile of monthly income, those in the middle tertile (aOR 4.28 95% CI 1.36, 13.53) and those in the lowest category of income (aOR 6.18 95% CI 1.83, 20.94) were significantly more likely to have poor treatment outcomes.

**Conclusion:**

We demonstrated that TB patients in Georgia with lower household income were at greater risk of poor TB treatment outcomes. Providing targeted social assistance to TB patients and their households may improve clinical response to anti-TB therapy.

## Background

Georgia, a country with a transitional economy, has one of the highest rates of tuberculosis (TB) in the European region including the former Soviet republics. In 2011, WHO estimated TB incidence in Georgia was 125 per 100,000 population (number of notified new and relapse cases was 4,547; TB prevalence was 159 per 100,000). WHO estimated high proportions of multi-drug resistant (MDR) TB cases (11% in new and 32% in previously treated cases) placing Georgia among 27 high MDR-TB burden countries [1].

The target for treatment success set by the Georgia TB Control Plan 2007–2011 was set at >85% [2]. However, the proportion of new smear positive cases achieving success remains low, in 2008 only 73% achieved this target, and in 2010 the proportion was 76% [1]. Treatment default remains one of the most important causes of poor TB treatment outcome for Georgian patients receiving anti TB therapy. In 2010, 7% of new TB patients with positive sputum smear or culture defaulted from TB treatment [1]. In the same year, default rate among MDR TB patients was reported as high as 21.9% [3].

Existing global epidemiologic evidence suggests an important association between various patient demographic and clinical characteristics (e.g. male sex, age ≥65 years, drug resistance, HIV co-infection, previously treated TB, cavitation) with poor TB treatment outcomes among new pulmonary TB patients [4-7]. In addition to the aforementioned factors, various social, behavioral, and economic characteristics (e.g. low education level, inadequate knowledge on TB, alcoholism, injection drug use) have been found to be associated with poor treatment outcomes including treatment default [8-10]. Previous studies have also shown low income and worsening socio-economic gradient over the course of illness may increase the risk of treatment default among TB patients [11,12]. There is also some evidence that stigma associated with TB may also contribute to treatment default among TB patients [13].

Whether household factors are associated with poor treatment outcome among newly diagnosed patients with pulmonary TB is not known in Georgia and has not been well studied. To date, no known published studies have used a prospective cohort design to examine household factors associated with risk of poor TB treatment outcomes (including default) in Georgia. Thus, the purpose of this cohort study was to determine household factors associated with poor TB treatment outcome among newly diagnosed patients with pulmonary TB in Georgia. Secondarily, we aimed to estimate the adjusted association between poor TB treatment outcome and: 1) household income, and 2) perceived household stigma from TB disease.

## Methods

### Study setting and population

We conducted a prospective cohort study in Tbilisi (the capital of Georgia), Kutaisi (the second largest city in Georgia), and the rural Samegrelo regions. Eligibility criteria included all consecutive new smear positive pulmonary TB patients seeking care during June – November 2004 at the National TB Center and five TB dispensaries (specialized facilities providing outpatient care to TB patients) in Tbilisi, one TB dispensary in Kutaisi, and one TB dispensary located in Samegrelo regional center Zugdidi. All eligible TB patients were asked by health care facility staff for informed consent for themselves and their household members to take part in the study. The study protocol was reviewed and approved by Tbilisi State Medical University IRB (N3/03 – 04.07.03).

### Data collection

Eligible patients were visited by trained study interviewers at their homes, and administered face-to-face questionnaires with TB patients and one key informant from the TB patient’s household. Data on household characteristics included both structured and semi-structured open-ended questions. A follow-up household interview was conducted six months after the baseline visit. If key informants were not at home at the time of interview, interviewers returned to the households up to three times.

Clinical data on TB patients were collected at specialized health care facilities providing care to these patients. According to national TB program requirements, [2] all patients received direct observed treatment and were actively followed up by health care providers for 6 months. Clinical TB data after 6 months was recorded from medical case records kept in each specialized facility.

The primary study outcome was TB treatment result after six months of follow-up during anti-TB treatment. The outcome was defined by WHO criteria – *poor outcome* was defined as default (treatment interruption for two consecutive months or more), failure (AFB smear positive after 6 months), or death [1]. *Favorable outcome* was defined as cured or completed therapy.

The study obtained sufficiently detailed household social profile to enable aggregation of findings by different social parameters (ethnicity, gender, age, knowledge about TB and a health belief and subjective experience of the illness). Socio-economic status indicators were also measured by obtaining self-reported estimates of household income, household expenditures, and health-related household expenditures (including payment for medical services, cost of transportation and medicines, and the financial burden of caring for patient). For this purpose, our study questionnaire used relevant sections of a survey instrument developed by the National Statistics Office of Georgia [14]. These data were used to calculate monthly household expenditure, including household healthcare expenditures.

The study also collected data on household perceived impact of TB, feelings of stigma, and extent of worry over TB as well as on household’s social support and networks, which was assessed by using relevant questions of World Health Survey 2002. Experiencing any stigma was defined as reporting any of the following (due to their TB disease): feeling as though they made others uncomfortable, feeling they had been treated inferior by others, or feeling that others had avoided contact with them [15].

### Statistical analyses

Statistical analyses were performed using SAS Version 9.3 (SAS Institute Inc., Cary, NC). Bivariate analyses were used to determine the association of individual patient and household level characteristics with poor TB treatment outcome. For bivariate analyses, the chi-square test was used to calculate p-values for categorical variables, and for continuous variables the Student’s t-test (for normally distributed) or Wilcoxon-Mann-Witney rank sums test (for non-normally distributed) was used. A two-sided p-value < 0.05 was considered statistically significant throughout the analyses. A multivariable logistic causal (non-elimination procedure) model was also used to estimate the adjusted association between patient household level characteristics and poor TB treatment outcome. Confounders included in the causal model were chosen based on directed acyclic graph theory and previous literature [16,17]. We used Eigenvalues, condition indexes, and variance decomposition proportions to identify variables that were co-linear in the multivariable model.

## Results

Among 303 patients with newly diagnosed pulmonary TB registered in Tbilisi (180), Kutaisi (63), and Samegrelo (60), 66.7% (202 of 303) of the cases were enrolled in the study (100 in Tbilisi, 51 in Kutaisi, and 51 in Zugdidi), and 95.5% (193 of 202) had complete data that was included in the analysis. Among enrolled TB patients, the median age was 35 years (inter quartile range [IQR] 21.0), 77.2%% were male, and the large majority (89.6%) were of Georgian ethnicity.

After six months of anti-TB treatment, 80.3% (155 of 193) had a favorable TB outcome (cured or completed). Among 38 (19.7%) patients with poor TB treatment outcome, 13 remained smear positive after 6 months of anti-TB treatment, 19 defaulted, (1 default was also smear positive), and 7 died.

Comparing TB patients with favorable treatment outcomes to those with poor treatment outcomes, patients with favorable outcomes were significantly younger (median age 33.0 vs. 42.5 years) and reported higher household income levels (median income 137 vs. 85 USD/month) at the time of TB treatment initiation (p-value <0.05, Table 1). Only 7 (4.5%) of patients with a favorable treatment outcome were AFB sputum smear positive after two months of anti-TB therapy compared to 10 (26.3%) among those with poor treatment outcomes (p-value <0.05). Patients with favorable TB outcomes more frequently reported some college education or greater (prevalence difference [PD] 7.6%, 95% CI -9.0%, 24.2%) and reported less unemployment (PD 8.7%, 95% CI -9.0%, 26.3% however, these differences were not detected at a statistically significant level.

**Table 1:** Baseline demographics, treatment characteristics, and household expenditures of TB patients with poor treatment outcomes, N = 193*

	**Poor TB treatment outcome**^**a**^	**Favorable TB treatment outcome**	**Total N (%)**
	**N (%)**	**N (%)**	**N = 193**
	**38 (19.7)**	**155 (80.3)**	
Age (years)			
Mean (STD)	42.4 (15.1)	36.1 (14.9)	37.4 (15.1)
Median (IQR)^c^	42.5 (26.0)	33.0 (21.0)	35.0 (21.0)
Sex			
Female	8 (21.1)	36 (23.2)	44 (22.8)
Male	30 (79.9)	119 (76.8)	149 (77.2)
Household ethnic group			
Georgian	36 (94.7)	137 (88.4)	173 (89.6)
Other	2 (5.3)	18 (11.6)	20 (10.4)
Residence location			
Rural	3 (7.9))	24 (15.5)	27 (14.0)
Urban	35 (92.1)	131 (84.5)	166 (86.0)
Education			
Some secondary	6 (15.8)	27 (17.4)	33 (17.1)
Completed secondary	20 (52.6)	68 (43.9)	88 (45.6)
Some college or greater	12 (31.6)	60 (38.7)	72 (37.3)
Month household income			
(USD)			
Mean (STD)	93 (79.6)	171 (155.2)	156 (146.7)
Median (IQR)^c^	85 (94.9)	137 (133.3)	127 (131.3)
0-89.9 USD/month^b,d^	18 (50.0)	41 (27.0)	59 (31.4)
90.0-162	13 (36.1)	47 (30.9)	60 (31.9)
>162	5 (13.9)	64 (42.1)	69 (36.7)
Occupation			
Employed	10 (26.3)	66 (42.6)	76 (39.4)
Unemployed	18 (47.4)	60 (38.7)	78 (40.4)
Other^e^	10 (26.3)	29 (18.7)	39 (20.2)
**TB characteristics**			
TB disease, self-reported			
Not serious/moderate	14 (36.8)	75 (48.4)	89 (46.1)
Very serious	24 (63.2)	80 (51.6)	104 (53.9)
TB treatment location			
Nurse administered	19 (54.3)	82 (52.9)	101 (53.2)
Dispensary	12 (34.3)	56 (36.1)	68 (35.8)
Other	4 (11.4)	17 (11.0)	21 (11.0)
2 month AFB smear^b^			
Positive	10 (26.3)	7 (4.5)	17 (8.8)
Negative	28 (73.7)	148 (95.5)	176 (91.2)
**Household stigma due to TB**			
Others feel uncomfortable			
Yes	7 (18.4)	28 (18.1)	35 (18.1)
No	19 (50.0)	75 (48.4)	94 (48.7)
Others don’t know	12 (31.6)	52 (33.6)	64 (33.2)
Others treat inferior			
Yes	6 (15.8)	17 (11.0)	23 (11.9)
No	20 (52.6)	86 (55.5)	106 (54.9)
Others don’t know	12 (31.6)	52 (33.6)	64 (33.2)
Others avoid contact			
Yes	8 (21.1)	31 (20.0)	39 (20.2)
No	18 (47.4)	72 (46.4)	90 (46.6)
Others don’t know	12 (31.6)	52 (33.6)	64 (33.2)
Any stigma^f^			
Yes	8 (21.1)	34 (21.9)	42 (21.8)
No	30 (78.9)	121 (78.1)	151 (78.2)
Worry/concern among household			
A lot	33 (94.3)	127 (84.1)	160 (86.0)
Some	2 (5.7)	20 (13.3)	22 (11.8)
A little/not at all	0	4 (2.6)	4 (2.2)
**Household expenditures (all figures in USD)**			
	**Household expenditures before TB treatment**		
Past month total, self-reported			
Mean (STD)	300 (474.1)	283 (208.7)	286 (279.2)
Median (IQR)	182 (181.8)	242 (212.1)	242 (193.9)
Past month total, calculated			
Mean (STD)	316 (399.3)	345 (253.4)	339 (287.0)
Median (IQR)^C^	236 (174.7)	303 (235.2)	283 (235.9)
Past month healthcare			
Mean (STD)	80 (93.4)	101 (105.1)	97 (103.0)
Median (IQR)^C^	48 (57.6)	67 (93.9)	61 (90.9)
Proportionate monthly income spent healthcare expenditure			
Mean	1.52 (2.27)	0.86 (1.04)	0.99 (1.38)
Median	0.75 (1.31)	0.53 (0.76)	0.56 (0.81)
<0.50	13 (36.1)	67 (44.1)	80 (42.6)
0.50-1.0	11 (30.6)	46 (30.3)	57 (30.3)
>1.0	12 (33.3)	39 (25.7)	51 (27.1)

*193 of 202 case patients had information available on treatment outcome.

^a^Defaulted, failed (AFB smear positive after 6 months), or death.

^b^Two sided Chi-Square p-value <0.05.

^c^Two sided Wilcoxon Rank sum p-value <0.05.

^d^Three month average income from waves 1–3.

^e^Other: student, retired, or homemaker.

^f^Combined variable: any yes response from *others feel uncomfortable, others treat inferior, or others avoid contact.*

Patients in this study commonly reported household stigma attributed to their TB disease. Overall, 21.8% (95% CI 16.4, 28.0) of patients with TB in the study reported experiencing any stigma due to TB (Table 1). Although not statistically significantly different, patients with poor TB treatment outcome more frequently reported feeling that others treated them inferior (84.1% vs. 94.3%, p-value = 0.71) and reported more worry or concern (94.3% vs. 84.1%, p-value = 0.47) compared to patients with favorable treatment outcome. Patients who experienced any stigma were (non-significantly) more likely to be AFB smear positive after two months (data not shown: OR 2.12 95% CI 0.74, 6.12).

### Results of multivariable analysis

In multivariable analysis adjusted for sex, age, household income, proportionate monthly income spent on healthcare, and stigma, only low baseline household income remained statistically significantly associated with poor TB treatment outcome (Table 2). A dose–response relationship was observed between household income and poor TB treatment outcome—compared to patients from households with the highest tertile of monthly income, those in the middle tertile were significantly more likely to have poor treatment outcomes (aOR 4.28 95% CI 1.36, 13.53), while those in the lowest category of income had the greatest odds of poor TB treatment outcome (aOR 6.18 95% CI 1.83, 20.94). Although not detected at a statistically significant level in the multivariable model, older patients were at increased odds of poor treatment outcome, while those patients with highest household proportionate income spent on healthcare were at decreased odds of poor TB treatment outcome. We did not detect a meaningful difference in the odds of poor TB treatment outcome comparing patients who reported experiencing any TB-related stigma compared to those who reported experiencing no stigma.

**Table 2:** Multivariable analysis for odds of poor treatment outcome among TB patients, N = 193

	**OR (95% CI)**	**AOR (95% CI)**^**a**^
Male	1.13 (0.48, 2.69)	1.02 (0.40, 2.70)
Female	1	1
Age (years)		
≥ 56	2.96 (0.95, 9.24)	
26-55	1.62 (0.70, 3.72)	
≤ 25	1	
Age (years)		
Per 1 year increase	1.03 (1.00, 1.05)	1.02 (1.00, 1.05)
Education		
Some secondary	1.05 (0.39, 2.81)	--
Completed secondary	1.47 (0.66, 3.26)	
Some college or greater	1	
Occupation		
Unemployed	1.98 (0.85, 4.63)	--
Other	2.28 (0.86, 6.06)	
Employed	1	
Household income		
0-89.9 USD/month	6.26 (2.20, 17.75)	6.18 (1.83, 20.94)
90.0-162	4.42 (1.45, 13.49)	4.28 (1.36, 13.53)
>162	1	1
Baseline household health care expenditures		
0-50 GEL/month	1.91 (0.69, 5.30)	--
51-200	1.19 (0.48, 2.97)	
>200	1	
Proportionate monthly income spent healthcare expenditure		
>1.0	1.59 (0.66, 3.82)	0.61 (0.21, 1.77)
0.50-1.0	1.23 (0.51, 2.99)	0.68 (0.24, 1.92)
<0.50	1	1
Any stigma		
Yes	0.95 (0.40, 2.26)	0.82 (0.30, 2.27)
No	1	1

^a^Adjusted model included sex, age, household income, health care expenditures, and proportionate monthly income spent on healthcare.

## Discussion

The main focus of this study was to examine the association between various household factors on 6-month TB treatment outcomes among newly diagnosed patients with pulmonary TB in the former Soviet Republic Georgia. In our cohort of patients, household income was significantly associated with poor TB treatment outcome after 6 months of therapy. The overall TB treatment success rate (80.3%) was below WHO recommended target of 85% but the proportion with successful outcome in our study was slightly higher than the nationwide treatment success rate of 73% (60% cured, 13% completed) reported by National TB Program (NTP) in 2005 [1].

Consistent with our results, several previous studies have demonstrated the negative impact of poor socio-economic conditions on TB treatment outcomes in both developing and developed countries. A study conducted in Estonia in 2005 found that unemployment was a significant predictor of TB-related deaths [18]. Similarly, a German study from 2001 found homelessness and unemployment to be strongly associated with non-cure of TB [19]. The main risk factor for death due to TB was having less than 6 years of formal education in Mexico [20]. Being urban resident was associated with higher treatment success rate in Ethiopia after adjusting for potential confounders [21]. In the same country, the odds of unsuccessful treatment outcome was significantly higher among patients with family size greater than 5 persons [22].

Prospective design of the study (i.e. collecting data on exposure at the baseline and assessing TB treatment outcome after 6 months of clinical follow up) allows delineating a negative role of low income in contributing to poor TB treatment outcome. However, nature of the data collected as well as type of the analysis conducted in this study was not robust enough to distinguish between direct and indirect effects of expose, i.e. a low socio-economic status on TB treatment outcome [23]. Accumulated psycho-social stress may be one plausible biological mechanism explaining the increased odds of poor TB treatment outcome among patients with low socio-economic status. Excess stress related to financial instability may deteriorate immune functions through long-term stress effects on the Hypothalamic-Pituitary-Adrenal axis, resulting in poor TB treatment outcome [24,25]. Another pathway though which low socio-economic status may influence treatment outcome may be through its negative impact on nutrition [26]. Improved nutrition is associated with increased strength of immune system in both human and animal studies, [27] and improved clinical outcomes among TB patients [28].

Our study is subject to important limitations. One methodological concern of this study was sampling: patients were recruited at specialized TB facilities in two major cities and in only one rural region. Consequently, the results may be more representative of TB patients living in urban areas, i.e. those with better socio-economic status or higher education [29]. Similarly, our sample size was relatively small and consequently power to detect statistical significance between examined associations was limited. Another limitation was inability to measure important clinical characteristics associated with poor TB treatment outcome (such as MDR-TB status and HIV co-infection). This data was not available because screening for MDR-TB during first 6 month of treatment was not part of routine clinical practice within the framework of NTP at the time of this study. Similarly, HIV status was not routinely recorded in TB patient case records. However, this might not seriously affect the study findings – based on NTP data, both proportion M/XDR-TB cases and TB-HIV co-infection rate among new TB cases have been 6.8% and 1.0%, respectively [30,31]. Based on the above considerations, we believe that a sufficient amount of this data can be generalized to new pulmonary tuberculosis patients in Georgia, although we recognize that the results of this study should be interpreted with some caution.

## Conclusion

In conclusion, data from our study, in addition to previous epidemiologic findings, strongly suggest that poor socio-economic conditions are negatively associated with TB treatment outcomes. We did not detect a strong association between perceived stigma due to TB disease and poor TB treatment outcome. Findings from our study suggest that the relationship between poverty and poor TB treatment outcomes is also an important public health concern in the country of Georgia. Improving targeted social assistance to TB patients and households with lower socio-economic resources may importantly reduce the risk of poor TB treatment outcomes.

## Abbreviations

(AFB): Acid-fast bacilli; (AOR): Adjusted odds ratio; (CI): Confidence interval; (XDR): Extensively drug-resistant; (GEL): Georgian lari; (HIV): Human Immunodeficiency virus; (IQR): Interquartile range; (IRB): Institutional review board; (MDR): Multi-drug resistant; (NTP): National TB program; (OR): Odds ratio; (SD): Standard deviation; (TB): Tuberculosis; (WHO): World Health Organization.

## Competing interests

The authors declare that they have no competing interests.

## Authors’ contributions

MD and EM conceived the research and study design, and drafted the manuscript. MJM led the analysis and co-edited the manuscript, NM assisted with data management and drafting the manuscript. All authors reviewed and approved the final manuscript.

## Pre-publication history

The pre-publication history for this paper can be accessed here:

http://www.biomedcentral.com/1471-2458/14/88/prepub
